# A Practical Treatise on the Diseases of the Lungs and Heart, Including the Principles of Physical Diagnosis

**Published:** 1852-02

**Authors:** 


### BIBLIOGRAPHICAL NOTICES.

*A practical Treatise on the Diseases of the Lungs and Heart, including the Principles of Physical Diagnosis.* By WALTER HAYLE WALSHE, M. D., Professor of the Principles and Practice of Medicine, and of Clinical Medicine, in University College, London; Physician to the University College Hospital, &c. 12mo. pp. 512. Philadelphia: Blanchard & Lea, 1851.

Most of our readers are, doubtless, familiar with the Essays which have placed Professor Walshe's reputation so high on the list of the scientific medical writers of Great Britain—his treatise on Cancer, and on "Adventitious Products," the last published in the Cyclopædia of Anatomy and Physiology. His public claims to practical skill in the diagnosis and treatment of the class of diseases to which the volume whose title we have above copied refers, have hitherto chiefly rested upon his Clinical Lectures, published in the Lancet, a report on Phthisis in the Brit. and For. Med. Rev., and a little book entitled "The Physical Diagnosis of diseases of the Lungs," published in 1842. This latter was reprinted in this country, in 1843, and found great favor with the profession. The present volume has a much wider range than its predecessor, as is evident from its different entitulation. We propose to exhibit to our readers its plan and scope.

Part 1st.—The first chapter treats of the physical examination of the Lungs,—by means of Inspection, application of the hand, Mensuration, Percussion, Auscultation, Succussion, and by determination of the situation of surrounding parts and organs. The second chapter is occupied with the physical examination of the Heart and great vessels, conducted by methods similar to those employed in the examination of the lungs.

Part 2d.—Applies these principles of investigation to the study of the diseases of the Lungs, Heart, and Aorta ; each disease of these organs respectively being separately considered in sufficient detail.

The volume is pretty equally divided between these two portions.

Many persons suppose that the recognition of diseases of the chest by physical exploration is a very simple and easy process; one which requires only the hearing ear, and the seeing eye. But this is a great mistake; and it is an error, too, which has given rise to very many wrong diagnoses, and brought much discredit on the method pursued. Others ridicule the whole procedure. Some years ago, we heard an eminent professor of the Theory and Practice of Medicine, who was at times facetious, assure a large class of students that it was nonsense to attempt to ascertain the existence of disease in the chest by the use of the stethoscope. Said he, "you might as well study astronomy with the aid of a clyster-pipe." Nor was the professor far astray, if the examiner rely only upon his ear, and not also upon his reasoning faculties.

There is no branch of practical medicine in which there is greater necessity for the exercise of sound judgment, careful discrimination, and logical inference, than this. The mind must be trained and educated, as well as the senses of hearing, touch and sight, before a physician can be considered a reliable investigator of the diseases of the thoracic organs.

The perusal of Dr. Walshe's treatise, will abundantly satisfy any one of these truths. We do not observe anything particularly novel in the plan which the author has adopted; but he exposes in a very striking manner the difficulties and sources of error which may perplex and mislead the examiner. Indeed, we do not remember to have seen in any other treatise on this subject, these points so well set forth. And in like manner his explanation of the difficulties, and of the means by which they may be overcome, and a correct opinion arrived at in spite of them, is equally satisfactory.

We shall now glance at a few of the prominent topics discussed.

After describing the method of conducting an examination of the chest by simple inspection, the application of the hand to its different regions, and mensuration, the author alludes to a means of diagnosis as to the amount of disease in the lungs, which has at different times attracted considerable attention, viz., by measuring the cubic inches of air which can be expelled from the chest by the fullest possible expiration, succeeding the fullest possible inspiration.

The instrument contrived by Dr. Hutchinson, which he terms the *spirometer*, is unquestionably the best adapted to ascertain this fact. By means of this apparatus, it will be recollected, this gentleman has ascertained that there exists a general ratio of proportion between the volume of air which the lungs in health are capable of containing, and the height of the individual. The mean quantity is 174 cubic inches for the height of 5 feet; and for every additional inch of stature from five to six feet, eight cubic inches of air, at the temperature of 60° F., are expelled by a forced expiration. Dr. Walshe admits the correctness of this law, in the main; but he very properly objects to its specific accuracy in practice, on the ground that however truly it may coincide with general results, "the individual healthy standard occasionally varies very widely on either side of the general one; so much so, that a great fall may have taken place, from disease, in the breathing-volume of an individual, at a time when he expels a quantity of air above the average standard of men above his height; according to the *general* standard he is more than healthy, he is extra-capacious; according to *his own*, he is diseased. For certainty of observation the individual standard is required; the present man must be compared with the past man, and not with other men. Again, a fall below the general average, is a surer iudication of disease than the maintenance of the general average, or even a slight excess, is of health." During the last few years, we have had the opportunity of testing pretty fully the applicability of the spirometrical guage; and we can most heartily agree with Dr. Walshe in the necessity of comparing the cubic capability of the lungs of the individual in disease of these organs, with that of the same in health, before any reliable opinion can be formed as to the existence or non-existence of pneumonic disease. And even then we do not think that we have any sufficient warranty for concluding that the reduced respiratory volume indicates affection of the respiratory organs; the difficulty may be entirely exterior to these, and seated in the moving powers of respiration,—in the spinal cord or nerves—or it may be due to imperfect development of the respiratory muscles.

The chapter on Percussion is very thoroughly studied, and contains a full statement of all the important details respecting this means of diagnosis.

The chapter on Auscultation as applied to the respiratory organs, occupies rather more than eighty pages—a proportion of the whole volume, which is not too large, considering the importance of the subject. In it we find remarks appertaining to all the questions which are likely to arise in the mind of any one who is studying this matter; not only are general directions given with reference to the practice of auscultation, but all the instances, so far as we can recall them, are adduced in which the particular auscultatory phenomena are observed, together with the explanation of this occurrence. It may be objected that the author refines too much for the necessities of practice. But we do not think so. The mere fact that many may be confused by multiplicity of detail, and be led into mistakes rather than benefited thereby, is no argument against the propriety of adopting any arrangement of subdivisions and nomenclature which will enable the student to arrive at the whole truth. We are sometimes afraid, it would seem, that we may become too accurately informed.

With regard to some of the auscultatory signs, Dr. Walshe thinks the crepitant rhonchus of Pneumonia "occurs in the parenchyma of the lung itself, especially in those portions of it immediately contiguous to, and actually forming the walls of, the ultimate termination of the bronchi; and that its physical cause is the sudden and forcible expansion of that parenchyma, glued together, as it were, by exudation with which it is infiltrated. Each single crepitus or click would thus signify the expansion of a cell, and be produced by the unfolding of surrounding glutinous tissue necessary for that expansion." (p. 113.) This, it will be noted, is not the ordinarily admitted rationale. Another explanation which is at least equally satisfactory, fixes the seat of this rhonchus within the air-cells, and ascribes it to the separation of their agglutinated walls.

The peculiarities of the cavernous rhonchus, and the sources of fallacy connected with it, are well exposed.

In his observations on the adventitious sounds produced in diseases of the pleura, the author refers to the occurrence of friction sound in emphysema of the lungs without pleurisy. (p. 123.) Laënnec first stated the possibility of this coincidence, and indeed considered it as highly important in the diagnosis of emphysema; but most subsequent authorities have denied this connection. Dr. Walshe is inclined, however, from cases which he has witnessed, and others of which he possesses records, to agree with Laënnec so far as to admit that it does happen in some instances that a friction sound is occasioned in emphysema beneath the pleura, by which the latter is rendered irregular and rough.

Some curious remarks are made upon "pleural pseudo-rhonchi," (p. 126) showing that a sound similar to that of the sub-crepitant rhonchus may be produced entirely exterior to the lungs, in the pleura, or rather in fluid contained in the meshes of adventitious tissue existing upon the pleura, in the movements of this membrane duriug respiration.

We are compelled to pass over the remainder of this chapter, exceedingly rich though it be in details concerning the phenomena of vocal auscultation, if we may so speak; indeed, we do not know where else this subject is so thoroughly and lucidly discussed.

The chapter which follows, on the physical examination of the heart and great vessels, occupies about as much space as the last, and is equally well worthy of perusal. We can call attention only to a few points from amongst the many which are presented to us.

Dr. Walshe does not attempt to explain the manner in which the *impulse* of the heart is produced, but ascribes it, as is ordinarily done, to the systole of the ventricles, without giving us his reasons for the opinion. This theory is open to objections, and we regret that the accomplished author of the treatise under consideration has not favored us with a critical examination of the question. It seems to us that the weight of evidence is in favor of the theory which attributes the heart's impulse against the walls of the chest to the *diastole* of the ventricle, which is rendered forcible by the active contraction of the auricle whereby the ventricle becomes filled with blood. The arguments in favor of this view are briefly but clearly stated by Dr. Stillé in the American Journal of Medical Sciences, July, 1846, p. 174, and also in his excellent treatise on General Pathology; they are also detailed at greater length by Hardi et Béhier, "Pathologie Interne," p. 326, vol. 1. To these sources we would refer our readers. The facts upon which these arguments are based, are derived from observations of the motions of the heart of the frog, which is transparent, and which is not disturbed in its action by the interference with the function of respiration which is unavoidable when vivisection is practised upon a warm-blooded animal; from watching the movements of the heart of the human being, when, as has not unfrequently happened, the anterior parietes of the chest have been congenitally wanting over the cardiac region, or have been removed by accident; and finally, from study of the phenomena presented in certain pathological conditions of the organ. "When the heart of the frog, which is transparent, is examined, the apex is seen to be thrust forward at the same instant that all the dimensions of the organ are enlarged, and its color shows it to be distended with blood; while the apex is retracted simultaneously with the lessening of the size, and the disappearance of the color of the heart; in other words, the projection of the apex corresponds with the diastole, and its retraction with the systole of the ventricle." (Stillé's, Gen. Pathol. p. 319.) In a case reported by Cruveilhier, in which a child was born with a deficiency in the upper part of the sternum through which the heart projected, and in which life continued for nine hours, the contraction of the auricles was seen to be exactly synchronous with the diastole of the ventricles. During the ventricular systole, the heart contracted in every diameter, and the only portion of it which was thrust forward was the apex, and that with a slow spiral motion. The diastole of these cavities, on the contrary, was accomplished with rapidity and energy, "so that the hand which had been closed upon the heart was opened with violence." A case similar to this was reported by Dr. Robinson of Va., in the American Journal, Feb. 1833; and we have very recently read the account of another analogous to these, but we have, unfortunately, forgotten where it is recorded. Finally, M. Beau, (Archives Gén. de Médécine, Dec. 1835, p. 426; July 1841, p. 300,) Dr. Corrigan of Dublin, and Dr. Stillé (Am. Journ. loc. cit.,) have shown that the analysis of reported cases of hypertrophy of the heart establishes the fact that, in simple hypertrophy of the ventricles, of any degree, the impulse is diminished or entirely wanting, unless there be corresponding hypertrophy of the auricles. From these statements it is evident that the cardiac impulse cannot be admitted to depend upon the ventricular systole alone, as is taught by Dr. Walshe. If the view here advocated be the correct one, it must be allowed too, to modify the author's explanation of the mode in which the *first sound* of the heart is produced; for, certainly, the active contraction of the auricles and the rushing of the blood through the auriculo-ventricular orifices must generate a sound. And, inasmuch as the ventricular systole follows instantly upon that of the auricles, the sounds generated by the two acts, together with the violent impetus of the blood against the now tense tricuspid and mitral valves, and the rushing of the torrent through the aortic and pulmonary openings, will sufficiently account for the dull, prolonged first sound.

In the application of percussion to the heart, to ascertain its boundaries and exact situation, we are glad to see that Dr. Walshe recognizes the importance of the suggestion first made by Drs. Camman and Clark, of New York, in favor of using the stethoscope while percussion is practiced. This combination certainly makes the investigation more satisfactory in doubtful cases; but it requires considerable familiarity with the practical manipulation, to make it a convenient means of diagnosis.

Everything relating to the modification of the normal action and sounds of the heart produced by diseases, both of this organ and of organs exterior to it, is most instructively considered.

We now pass to that portion of the book which is devoted to the *Diseases* of the thoracic viscera. This, as we before remarked, occupies about one-half of the volume, a space not large enough, indeed, to enable the author to be as minute and thorough as his readers would desire; but we can assure them that his powers of compressing and condensing the results of his enlarged experience and logical study, have been exercised in a most remarkable manner. We shall ask attention to only a few topics.

His remarks on dilatation of the bronchial tubes are admirable. After alluding to the secondary effects produced by this abnormal condition, he thus points out the means of distinguishing it from phthisis. "In phthisis, percussion is dull *above* the clavicle; not so in dilated bronchus; below the clavicle, too, the dulness is greater and more extensive in the former than in the latter case. The signs of the tubercular excavation are found at the apex; those of dilated bronchus generally lower—say at the union of the middle with the upper third of the chest. When tubercle has reached the excavation stage, flattening at the surface is habitually more marked under the clavicle than it ever is in bronchial dilatation. I have never known hemoptysis produced by chronic bronchitis, with dilatation alone; if it exist, and there be no evidence of mitral disease, the evidence that the excavation is tuberculous, becomes a matter of necessity. Extreme emaciation does not, so far as I have seen, come of the bronchial disease alone. The course of the physical signs will avail us also, if the case continue for a time under observation. In phthisis, the signs are, as a rule, constantly increasing in degree and extent; in bronchial dilatation, they may remain for months unaltered in both these respects; dulness under percussion, as remarked by Dr. Stokes, precedes the signs of cavity in phthisis, and does not occur till after them in bronchial dilatation; to the latter clause, however, I have seen exceptions." Page 250.

The subject of Pleurisy, in all its varieties, is very ably treated. Concerning the *prognosis*, he says: "Death is so rare a result of the disease, when attacking individuals free from organic affections, that I have neither myself (and I have carefully attendedito this point, since my attention was first drawn to it, years ago, by M. Louis) lost a patient from pure primary idiopathic pleurisy, with or without effusion, nor known of an occurrence of the kind in the practice of others." (Page 269.) The treatment recommended for the acute stage consists in moderate depletion, general and local; slight mercurialization; and the application of a blister over the *neighborhood* of the chiefly affected part, rather than over the spot itself. If fluid accumulate, or remain long in the cavity of the chest, flying blisters in succession, and ioduretted liniments should be employed externally, and diuretics internally; among the latter, Dr. Walshe advises the tincture of iodine, in scruple doses, freely diluted.

The author's remarks concerning the propriety of *paracentesis*, the time of its performance, &c. are well conceived. He agrees with most recent authoritative writers, in recommending its early performance, after the physician has become satisfied that absorption has not taken place under proper treatment.

*Pneumonia*, as might have been anticipated, receives its full share of consideration. The author admits that in some instances, as when the closure of the bronchial tubes is very extensive, and the conducting powers of the lung are, in some inappreciable manner, annulled, the physical signs may be wholly wanting, excepting that of dulness on percussion; that simple pneumonia, without pleuritic effusion of any kind, may produce expansion, and, subsequently, contraction of the chest; that the old fashioned practice of very large and repeated bleedings is unwise and unnecessary; that tartar emetic is a remedy of vast importance in the treatment of this disease, and that "mercurials appear to him to be desirable in those cases only, where, for some cause or other, antimony is inadmissible." We cannot too highly commend this section of the volume, and we regret that our limits will not permit us to make copious extracts from it, for there is scarcely a question of interest which does not receive light from Dr. Walshe's observations.

The section on Phthisis is one of the ablest in the book, and this is a warm eulogium where all are good. Few physicians have had so extensive opportunities for studying this disease as the author of this treatise, and none are better qualified for the task. The points to which especial attention is herein devoted are, the significancy of hæmorrhage as a symptom of phthisis; the means of arriving at the diagnosis of the incipiency of this affection; and the efficacy of treatment in curing or mitigating it. He thinks that cod-liver oil "more rapidly and effectually induces improvement in the general and local symptoms than any other known agent," but that the degree and permanency of its curative powers, are not yet determined sufficiently; he prefers the *brown* oil to either of the other varieties. "The dose of the oil at the outset should never exceed, (often fall short of,) a drachm twice daily; it may be taken in milk, water, orange-wine, or any other aromatic water agreeable to the patient. I have never seen any good, but often ill, effects to follow the attempts to pour in large quantities." Page 360.

An interesting section is allotted by the author to the diagnosis of intra-thoracic tumor, which is of great consequence, as it is so easily confounded with aneurism.

Diseases of the heart and large vessels, occupy rather more than one hundred pages of the volume. We should like very much to review this portion of the book carefully, and to quote from it for the edification of our readers. But the details into which we have been tempted by our admiration of that part of the work which we have already noticed, make it necessary that we shall proportionably neglect this. It contains, notwithstanding, abundant food for study and profitable search. But we must here bring our remarks to a close, assuring those who may have followed us thus far, that the excellence of the treatise is the same to the very end. We can unhesitatingly recommend it as the best in the language.

